# Cost analysis of outbreaks with Methicillin-resistant *Staphylococcus aureus* (MRSA) in Dutch long-term care facilities (LTCF)

**DOI:** 10.1371/journal.pone.0208092

**Published:** 2018-11-26

**Authors:** Antonius M. van Rijt, Jan-Willem H. Dik, Mariëtte Lokate, Maarten J. Postma, Alex W. Friedrich

**Affiliations:** 1 Department of Medical Microbiology, University Medical Center Groningen, Groningen, The Netherlands; 2 Faculty of Medical Sciences, University of Groningen, Groningen, The Netherlands; Australian National University, AUSTRALIA

## Abstract

**Objectives:**

Highly resistant microorganisms (HRMOs) are of high concern worldwide and are becoming increasingly less susceptible for antibiotics. To study the cost effectiveness of infection prevention measures in long-term care, it is essential to first fully understand the impact of HRMOs. The objective of this study is to identify the costs associated with outbreaks caused by Methicillin-resistant *Staphylococcus aureus* (MRSA) in Dutch long-term care facilities (LTCF).

**Methods:**

After an outbreak of MRSA, Dutch LTCF can submit a reimbursement form to the Dutch Healthcare Authority (“Nederlandse Zorgautoriteit”; NZa) to get a part of the total costs reimbursed. In this study, we requested NZa forms for financial impact analysis. Details regarding the costs of the outbreak have been extracted from these forms and additionally specific LTCF have been visited in person to validate the data.

**Results:**

34 complete reimbursement forms from the period between 2011 and 2016 were received from the NZa and have been included. The median cost per patient per day was estimated at €83.80, varying between €16.89 and €1,820.09. We validated five reimbursement forms by visiting the facility and recalculating the costs. We found a non-significant positive difference of €26.07 compared with the original data (p = 0.068).

**Conclusions:**

This study is to our knowledge the first to give a national overview of total costs associated with an MRSA outbreak in LTCF in the Netherlands. Overall, costs per patient per day seem lower than in a hospital setting, although total costs are much higher due to the long term of care.

## Introduction

Due to the use of antibiotics, treatment options for infections caused by microorganisms are decreasing. Microorganisms that once were susceptible, have now become resistant to most of the commonly used antibiotics [1]. Highly resistant microorganisms (HRMOs) are starting to spread over the world, creating a huge clinical and financial burden, making infection prevention measures more important than ever before [1–3]. Especially in hospitals in the Netherlands, infection prevention measures are well implemented [4,5]. However, resources directed to infection prevention/control in long-term care facilities (LTCF) lag behind [6]. In LTCF, five perceived barriers to infection prevention were identified: ‘language and culture’, ‘knowledge and training’, ‘per-diem and part-time staff’, ‘workload’ and ‘accountability’ [7]. Outbreaks with HRMOs also occur in LTCF, which are often even harder to combat due to long-term care patients and mutual contact between patients [8]. However, the costs of these outbreaks are often unknown. To create awareness of the positive effects of infection prevention management, and to properly study cost effectiveness, it is first necessary to study what the actual costs of an outbreak in a LTCF are and to communicate these to underpin the seriousness of the issue.

Difficult to treat infections occurring in LTCF that are caused by HRMOs pose a big problem, not just for LTCF, but for hospitals as well. LTCF, even those that are not connected to a hospital by direct patient transfers, play an important role in the transmission of HRMOs in a specific area and can thus be key in regional and hospital infection control strategies [9,10].

The prevalence of HRMOs in Europe differs between countries [2]. The Netherlands is one of the few countries in Europe that actively and successfully tries to prevent the spreading of HRMOs. Thanks to consequent implementation of focused policies, like the “search-and-destroy” policy for methicillin-resistant *Staphylococcus aureus* (MRSA) and other factors related to the structure of the healthcare system, the rates of MRSA and other HRMOs in the Netherlands are relatively low, compared to other countries in Europe and beyond [2,11,12]. In the Netherlands, the prevalence of MRSA in nursing homes varies between 0–1% [12–14]. Treatment of such infections is becoming more complicated and high costs of treatment are inevitable [1,15].

After admission, many patients remain in the LTCF for the rest of their life. Comfort, a social environment and preserving the functional status of patients are main objectives for a long-term care facility, rather than discharge patients [16]. Ergo, when there is an outbreak of an HRMO, the duration will often be much longer than in a hospital setting, since patients are not leaving or at least stay very long. The elimination of an outbreak with a highly resistant microorganism is possible, however sometimes an outbreak takes years and ends when all the infected patients have passed away or decolonized in time [16,17]. There are several guidelines on infection prevention for employees of nursing and residential homes to minimize the chance of an outbreak. However, once in a while an outbreak will occur. To contain and eliminate the outbreak, substantial amounts of funds and resources are used. These amounts increase even further when the outbreak is left unnoticed at first [18,19].

Knowledge about the costs of outbreaks in LTCF would make it a lot easier for nursing and residential homes to make choices about specific forms of infection prevention protocols and control measures, especially since infection prevention measures are costly [20]. Therefore, it is necessary to identify different resources, manpower and costs that are associated with containing and eliminating an outbreak. With this information it can be analyzed whether the costs of infection prevention measures outweigh the costs associated with an outbreak. By analyzing processes, major costs and impacts of different microorganisms causing outbreaks, comprehensive cost totals can be identified. With this information, better decisions can be made regarding funding of infection prevention, screening and control measures. Ultimately, enhanced prevention will lead to decreasing the chance of another outbreak and minimizing the spread of highly resistant microorganisms.

Since 2005, Dutch LTCF can get the costs made for outbreaks with MRSA partly reimbursed from the Dutch Healthcare Authority (Nederlandse Zorgautoriteit [NZa]). Through this procedure, a lot of data is available on these outbreaks in the standardized forms used for this purpose. This study aims to analyze these data on the costs of MRSA outbreaks with the explicit objective to identify the costs associated with multiple outbreaks caused by MRSA in Dutch LTCF.

## Methods

On behalf of the government, the NZa facilitates reimbursements for MRSA outbreaks in LTCF. For this purpose, a form is filed by the facility to the NZA comprising an inventory of initiated actions and costs. Data from all reimbursements between January 2011 and December 2016 were requested and kindly provided by the NZa. Only the forms that were a 100% complete have been included. The data from these reimbursement forms comprise the national data used in this study. There are fixed categories for costs and, since every outbreak is unique, sometimes not all costs can be accommodated in the forms. Also, facilities interpret the form in their own way. As it is therefore not exactly known how these reimbursement forms compare to reality, a subset of five facilities from the national data was included in an on-site audit to validate the reimbursement forms.

This research has been carried out according to the Dutch guideline for conducting economic evaluations in healthcare where applicable [21,22]. All data used was anonymous and researchers could not identify individual patients or employees. The data processing was approved by the local data protection commissioner of the University Medical Center Groningen and the Medical Ethics Review Board of the University Medical Center Groningen waived approval (ref. nr.: 2016/531).

### Forms

The healthcare authority compensates a facility for the costs made by an outbreak with MRSA. These costs can include several posts: medical microbiology, antimicrobial resources, personnel- and material, personal protective equipment, cleaning and costs regarding the closure of a facility or certain departments of a facility. The reference price for closure varies per year and is set by the NZa. All other costs are filled out on a reimbursement form by the facility itself and are based on the facilities actual expenses. The reimbursement forms are checked by an external committee, which advises the healthcare authority on acceptability before reimbursements are made. 1% of the accepted costs, to a maximum of €25,000, must be paid by the facility itself.

### On-site audit

All facilities included in the national dataset were contacted and asked if they would participate with an on-site visit to establish the real costs of an outbreak regardless of the data submitted in the reimbursement forms.

All identifiable extra costs made by the LTCF caused by the outbreak were included and analyzed from the LTCF perspective. This contrasts with the reimbursement forms submitted at the NZa, where facilities can only fill in certain costs that fit into the pre-specified categories. Therefore, the on-site audits potentially include costs that are not mentioned in the reimbursement form. The start of an outbreak was defined as the day at which several carriers with MRSA were recognized. The end of the outbreak was the day at which no further measures were in place to control the outbreak. Every included outbreak has been checked by the NZa and has been confirmed as an outbreak. Costs incurred by the LTCF due to an outbreak have been included till one year after the end date.

Costs were divided into separate categories, namely: microbiological diagnostics, personnel, materials, cleaning and disinfection, missed income due to closed beds and other costs. Patient isolation was not included as a specific category. Costs for isolation, such as specific materials, fit into the previously mentioned categories.

All relevant documents and financial information from the facilities were gathered, to analyze what extra resources were used to control and eliminate the outbreak. Different sources for data were used to make sure no costs were overlooked. The additional amount of cultures and other microbiological diagnostics were acquired in corporation with the local laboratory. Interviews with managers, nurses and other relevant personnel took place to calculate personnel costs. Also, opportunity costs for personnel were included. Additional costs for materials were based on interviews and documentation, including personal protective equipment for personnel and prescribing of extra medication and antimicrobial agents. The extra costs made for cleaning were based on documentation and invoices. Missed income was calculated per empty bed per day in comparison with normal occupation. The total number of empty bed days was multiplied with the reference price (€168) set by the Dutch guideline for conducting economic evaluations in healthcare and is corrected for inflation [21,22]. If there was no extra information available on certain costs or for example the total hours of meetings, the amounts were estimated in consultation with the local manager of the facility and an expert on infection prevention. Costs that could not be specified into different categories were distributed over all categories according to the percentage distribution of the remainder of the costs that could be classified. In the results, costs are consistently tabulated according to the categorization described. In the end, the total amount per category for the national data and the on-site audit has been adjusted for inflation to 2015 price levels by using the Dutch consumer index figures [23]. If an outbreak took place over several years, the first year of the outbreak was used as reference point to adjust the costs for inflation.

### Calculation

For the financial analysis, the total costs for all categories of the different outbreaks were calculated. If the data was not normally distributed, the median and range were used.

The national data and the data from the on-site audit were compared with a Wilcoxon Signed Rank Sum Test. A sensitivity analysis was performed to see what the total costs would be if the reference price for missed income would be 25% lower or higher. Calculations were performed with Microsoft Excel (Microsoft, Redmond, WA, USA) and SPSS (IBM, Amonk, NY, USA). A significance level of p <0.05 was applied to test statistical significance.

## Results

### Total costs per patient per day ranged between €16.89 and €1,820.09

In total, 46 reimbursement forms from the period between 2011 and 2016 were received from the NZa. Of these, 34 forms were complete and were analyzed. Table 1 shows the characteristics of these outbreaks. The costs ranged between €16.89 and €1,820.09 per patient per day (median €83.80). Five outbreaks were included in the on-site audit and four are marked with an extra number behind the assigned number. This extra number equals the number of the outbreak in the on-site audit. The reimbursement form that corresponds with outbreak number two in the on-site audit was incomplete and therefore not included in the national data. Table 2 shows an overview of the characteristics of the outbreaks included in the on-site audit. The costs ranged between €14.02 and €545.24 per patient per day (median €109.87).

**Table 1:** Characteristics of the national data.

Nr.	Year	Positive patients	Positive personnel	Duration (days)	Total costs per patient per day	Total costs of outbreak
1	2014	14	2	30	€107.73	€45,244.58
2	2015	5	3	19	€439.65	€41,767.11
3	2015	10	2	40	€134.71	€53,882.80
4	2013	8	0	134	€110.43	€118,379.57
5	2014	27	7	466	€21.57	€271,442.39
6 ^5^	2015	20	9	129	€103.28	€266,471.96
7	2014	8	4	100	€79.73	€63,786.60
8	2015	18	17	56	€266.95	€269,085.41
9 ^4^	2015	79	0	331	€37.32	€975,879.75
10	2014	21	7	101	€82.88	€175,797.80
11 ^3^	2014	43	3	161	€60.49	€418,783.67
12	2014	11	1	47	€70.27	€36,328.13
13	2014	17	7	44	€84.71	€63,364.11
14	2014	31	1	365	€16.89	€191,138.24
15	2014	6	0	350	€23.27	€48,872.08
16	2012	36	19	202	€64.80	€471,216.42
17	2013	13	0	365	€59.97	€284,570.11
18	2013	51	9	159	€37.18	€301,514.90
19	2013	6	1	100	€101.69	€61,015.97
20	2013	20	26	286	€68.08	€389,417.35
21	2013	9	3	293	€48.68	€128,368.66
22	2012	3	3	129	€291.63	€112,859.24
23	2012	2	0	51	€607.81	€61,996.31
24	2012	1	1	33	€1,820.09	€60,062.90
25	2011	21	7	382	€36.89	€295,912.83
26	2011	16	1	88	€78.32	€110,278.19
27	2011	3	1	14	€1,100.91	€46,238.01
28	2011	8	5	109	€62.84	€54,793.33
29	2011	5	4	49	€394.15	€96,567.02
30	2011	1	0	49	€70.19	€3,439.35
31	2011	4	2	112	€133.30	€59,716.54
32	2011	8	5	151	€213.92	€258,420.04
33 ^1^	2011	8	6	39	€471.50	€147,106.74
34	2011	10	3	29	€169.05	€49,025.20

^1, 3, 4, 5^: Correspond with outbreak 1, 3, 4 and 5 in Tables 2 and 4.

**Table 2:** Characteristics of the on-site audit.

Nr.	Long-term care facility	Year	Positive patients	Positive personnel	Duration (days)	Total costs per patient per day	Total costs of outbreak
1	Facility 1	2011	8	6	39	€545,24	€170.113,72
2 *	Facility 2	2012	50	26	1676	€14,02	€1.174.829,45
3	Facility 3	2014	43	43	161	€109,87	€760.632,38
4	Facility 3	2015	79	79	331	€74,78	€1.955.387,16
5 **	Facility 4	2015	20	9	164	€129,32	€424.170,34

*: Outbreak is not eliminated at time of investigation

**: Outbreak includes €70.000 unspecified costs made in 2016, divided over all categories.

### Total costs on-site audit higher than national data but non-significant

**Fig 1:**
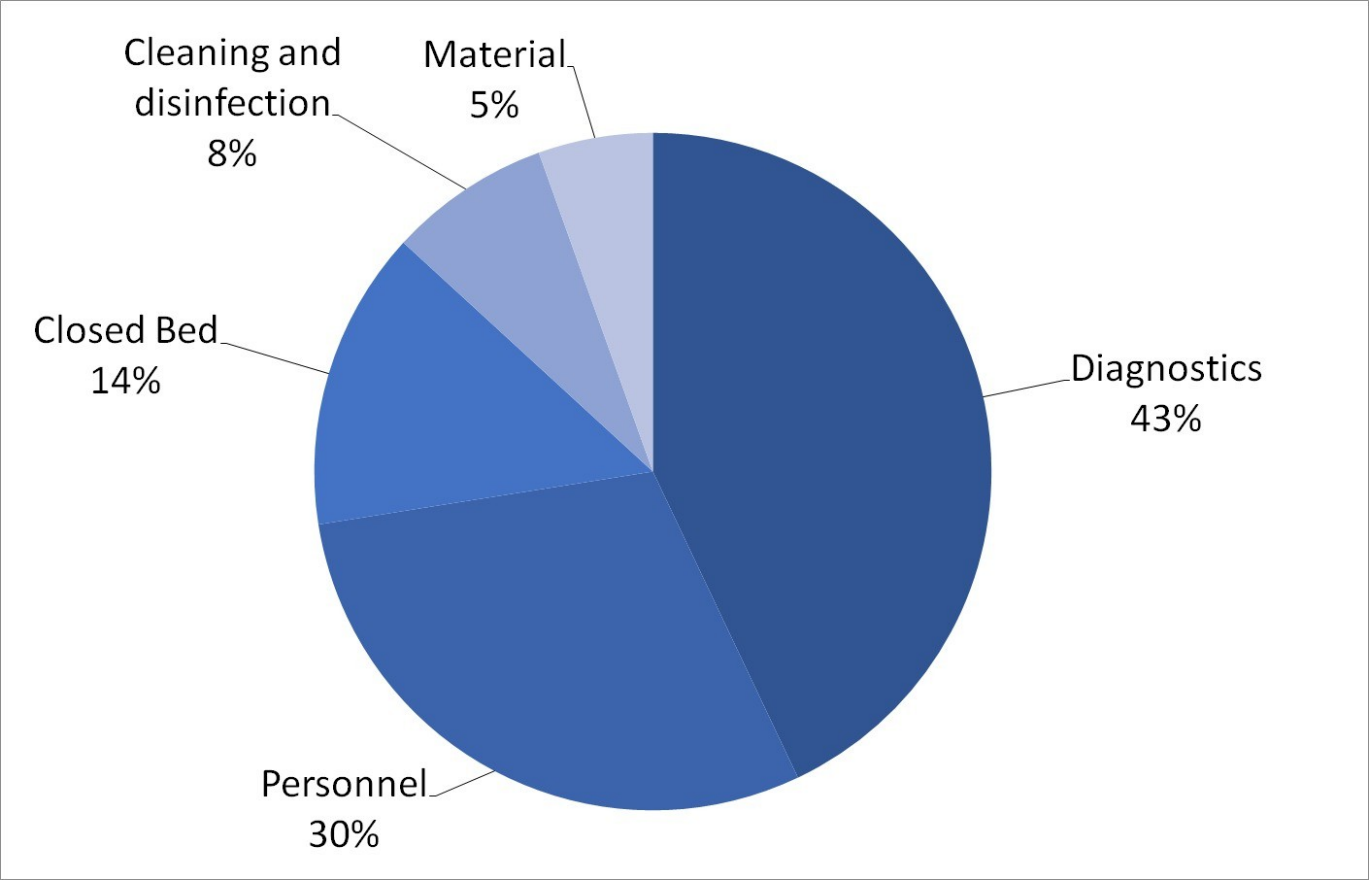
Median costs of outbreaks with MRSA in the national data.

Table 3 shows the costs per patient per day for the outbreaks included in the national data and Table 4 shows these costs for the on-site audit. The distribution of the costs among different categories are shown in Fig 1. For the national data most costs are made by diagnostics (43%). The second largest expense concerns personnel (30%).

**Table 3:** Costs per positive patient per day in the national data.

Nr.	Diagnostics	Personnel	Material	Cleaning and disinfection	Closed Bed	Total
1	€20.00	€29.99	€6.25	€46.29	€5.19	€107.73
2	€88.30	€150.38	€77.81	€123.16	€0.00	€439.65
3	€51.69	€62.86	€6.50	€7.16	€6.48	€134.71
4	€52.26	€50.18	€3.99	€2.67	€1.33	€110.43
5	€8.95	€7.74	€1.06	€1.97	€1.85	€21.57
6 ^5^	€14.67	€23.53	€9.54	€33.21	€22.34	€103.28
7	€9.28	€22.64	€5.69	€6.14	€35.99	€79.73
8	€58.43	€23.66	€10.64	€6.18	€168.05	€266.95
9 ^4^	€11.58	€10.12	€2.18	€2.10	€11.35	€37.32
10	€33.73	€30.68	€12.21	€3.87	€2.40	€82.88
11 ^3^	€17.73	€16.20	€8.32	€9.93	€8.31	€60.49
12	€50.60	€2.64	€4.06	€6.64	€6.33	€70.27
13	€45.54	€6.59	€3.45	€8.24	€20.90	€84.71
14	€2.97	€0.48	€3.91	€7.55	€1.99	€16.89
15	€4.28	€0.26	€5.72	€13.02	€0.00	€23.27
16	€34.85	€16.65	€6.30	€2.21	€4.79	€64.80
17	€5.97	€41.20	€1.13	€0.70	€10.98	€59.97
18	€9.66	€5.11	€5.53	€7.83	€9.06	€37.18
19	€18.82	€48.17	€4.09	€20.74	€9.87	€101.69
20	€35.21	€12.19	€2.01	€0.00	€18.67	€68.08
21	€21.28	€10.65	€3.07	€2.18	€11.50	€48.68
22	€98.20	€132.12	€0.00	€37.49	€23.82	€291.63
23	€403.68	€151.59	€5.31	€1.33	€45.90	€607.81
24	€543.57	€1,241.13	€16.96	€3.16	€15.28	€1,820.09
25	€15.26	€10.18	€0.44	€2.97	€8.03	€36.89
26	€8.48	€34.62	€2.43	€3.88	€28.91	€78.32
27	€383.31	€119.55	€20.99	€41.19	€535.87	€1,100.91
28	€17.16	€6.67	€0.33	€5.06	€33.62	€62.84
29	€154.23	€67.99	€59.42	€66.58	€45.93	€394.15
30	€64.53	€5.66	€0.00	€0.00	€0.00	€70.19
31	€40.87	€52.97	€4.64	€1.72	€33.09	€133.30
32	€19.89	€153.95	€3.16	€12.66	€24.27	€213.92
33 ^1^	€102.89	€174.19	€21.82	€159.20	€13.39	€471.50
34	€74.23	€0.00	€1.42	€0.00	€93.41	€169.05

^1, 3, 4, 5^: Correspond with outbreak 1, 3, 4 and 5 in Tables 2 and 4.

**Table 4:** Costs per positive patient per day for outbreaks included in the on-site audit.

Nr.	Long-term care facility	Diagnostics	Personnel	Material	Cleaning and disinfection	Closed Bed	Total
1	Facility 1	€108,13	€238,41	€30,42	€133,01	€33,59	€545,24
2 *	Facility 2	€4,24	€5,01	€1,53	€0,46	€2,78	€14,02
3	Facility 3	€17,73	€54,58	€8,32	€9,93	€19,31	€109,87
4	Facility 3	€13,00	€30,88	€2,18	€2,10	€26,62	€74,78
5 **	Facility 4	€13,82	€25,88	€13,49	€26,76	€49,37	€129,32

*: Outbreak is not eliminated at time of investigation

**: Outbreak includes €70.000 unspecified costs made in 2016, divided over all categories.

**Fig 2:**
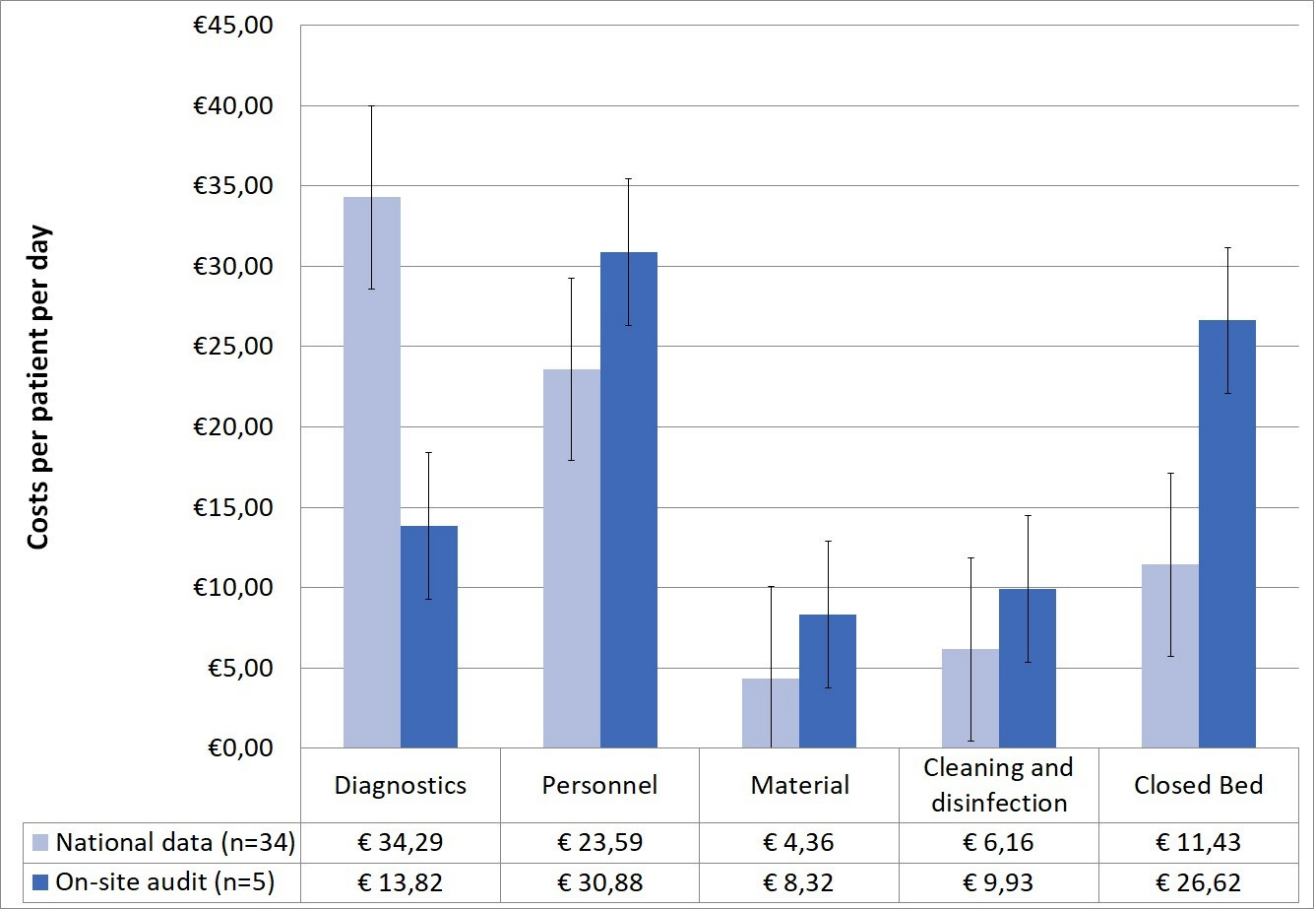
Median costs of MRSA, national data compared with on-site audit.

Since the data was not normally distributed, medians have been calculated. Table 5 shows that the median of the total costs is higher in the data from the on-site audit (€109.87 against €83.80 from the national data). The costs for diagnostics are higher in the national data and for all other categories the costs are higher in the on-site audit as is shown in Fig 2.

**Table 5:** Comparison of national data with the on-site audit (amounts per positive patient per patient day).

Data	N	Calculation	Duration (days)	Positive Patients	Positive Personnel	Diagnostics	Personnel	Material	Cleaning and disinfection	Closed Bed	Total	Total costs of outbreak
**National data**	34	Median (Range)	105 (14–466)	10 (1–79)	3 (0–26)	€34.29 (3–544)	€23.59 (0–1241)	€4.36 (0–78)	€6.16 (0–159)	€11.43 (0–536)	€83.80 (17–1820)	€111,568.71 (3,439–975,880)
**On-site audit**	5	Median (Range)	164 (39–1676)	43 (8–79)	6 (0–26)	€13.82 (4–108)	€30.88 (5–238)	€8.32 (1–30)	€9.93 (0–133)	€26.62 (2–49)	€109.87 (14–545)	€760,632.38 (170,113–1,955,387)

The total costs per patient per day from the national data have been compared with the data from the on-site audit. The four marked outbreaks that are included in the on-site audit and in the national data were not normally distributed and have therefore been compared with a Wilcoxon Signed Ranked Sum test. There was no significant difference between the total costs per patient per day from the four selected outbreaks in the national data (median €81.89) and the data from the on-site audit (median €109.87), however, there was a trend towards significance (p = 0.068, Z = -1.826).

### Sensitivity analysis shows no discrepancies between different reference prices

A sensitivity analysis with a 25% margin of the reference price was performed (Table 6). The sensitivity analysis shows no further discrepancies.

**Table 6:** Results sensitivity analysis of the on-site audit.

Nr.	Long-term care facility	Normal costs	25% Higher reference price	25% Lower reference price
1	Facility 1	€545,24	€553,63	€536,84
2	Facility 2 *	€14,02	€14,71	€13,32
3	Facility 3	€109,87	€114,70	€105,04
4	Facility 3	€74,78	€81,43	€68,12
5	Facility 4 **	€129,32	€141,66	€116,98

Reference price is per empty bed per day; Costs are per patient per day

*: Outbreak is not eliminated at time of investigation

**: Outbreak includes €70.000 unspecified costs made in 2016, divided over all categories.

## Conclusions

Literature on the costs of outbreaks with MRSA in long-term care is scarce. This research provides an overview of such costs in the Netherlands. The goal is to give decision-makers in LTCF evidence for better choice in the implementation of infection prevention and control measures. Total costs were divided into the separate categories of diagnostics, personnel, cleaning and disinfection, material and closed beds.

The costs found lie between €16.89 and €1,820.09 with a median of €83.80 per patient per day. Significant cost drivers are personnel, diagnostics and closed beds. Compared to outbreaks in the hospital setting, the costs per patient per day seem lower in LTCF. An on-site audit showed that the reimbursement forms might provide an underestimation of the costs and total costs might be even higher than presented here.

Further research is needed to investigate whether the costs of implementation of infection prevention measures outweigh the potential costs associated with an outbreak. With the current system, LTCF get the costs of an outbreak reimbursed, except for 1% of the acceptable costs with a maximum of €25,000. It is questionable if this refunding is enough to create a financial incentive for LTCF to improve their infection prevention measures. A prevention-driven incentive could be to reward facilities as long as no outbreak occurs, or to reimburse resources spend on infection prevention measures.

Controlling outbreaks requires a coordinated set of actions and stakeholders working together on diagnostics, infection prevention and antibiotic therapy. Nowadays, antimicrobial stewardship is hailed as one of the most important solutions of resistance. We argue that diagnostic stewardship and infection prevention measures are equally important, especially in LTCF where antibiotic therapy is more rare [24,25]. Only with an integrated approach of all three stewardship programs to infections and outbreaks, the most effective results can be achieved.

## Discussion

We included 34 outbreaks in the Dutch national data in this study, that were subsequently analyzed. The completeness of the costs of these outbreaks has been validated by analyzing five outbreaks in an on-site audit. There was no significant difference found between the total costs based on the national data and the on-site audit, although there is a trend towards significance that shows that the reimbursed costs are lower than the actual costs (p = 0.068). However, this trend is probably caused by the outbreak included in the on-site audit that was not yet finished and was not included in the national data.

There is not much known about the costs of outbreaks in healthcare facilities, especially in long-term care. Dik et al. analyzed seven outbreaks in the hospital setting. One of these outbreaks was an outbreak with MRSA. They found a total cost per patient per day of €657.08 (2015 price level). This is higher than the median of the costs we found. However, the duration of the outbreak was only 16 days. At the beginning of an outbreak, a lot of costs are made. After a certain period, only the fixed costs will remain. This causes the total costs per patient per day to drop, but the total costs of the outbreak to keep rising. Therefore, it is not possible to compare outbreaks that occurred in a hospital with outbreaks that occurred in LTCF using total costs per patient per day. The total costs estimated by Dik et al amounted to €31,539.84. Compared with this result, the total costs in the LTCF analyzed here are much higher at €111,568.71 [26].

In 2003, Capitano et al. published an article comparing the costs per patient between MRSA and Methicillin-susceptible *Staphylococcus aureus* (MSSA) in a long-term care facility. The costs for MRSA were 1.95 times greater than the costs for MSSA at a median of $2,607 (range $849–8,895) versus MSSA at a median of $1,332 (range $268–7,265). Nursing care with more than 2 times higher costs in the MRSA group and infection management with more than 6 times higher costs in the MRSA group were the largest and second largest components of the total costs of the infection, respectively [27]. If we correct the outbreak with MRSA for inflation and convert it to 2015 price levels, the outbreak that Capitano et al. described would cost €2,798.83 [23,27,28]. Our result is substantially higher than as found by Capitano et al., but the number of positive patients was lower (Capitano et al.: 41, our national data: 16). Notably, the amounts described by Capitano et al. are based on one outbreak in a LTCF, and the duration of the outbreak was not reported.

The duration of outbreaks with MRSA differs between hospitals and nursing homes. Dik et al. describes an outbreak with MRSA in a hospital with a duration of 16 days and van der Bij et al. found an average duration of 40 days [26,29]. An outbreak in a nursing home in Norway took 20 months [30]. The duration of outbreaks in LTCF included in this study lie between 14 and 466 days.

Strength of this study is the fact that multiple outbreaks with MRSA nationwide have been evaluated. All LTCF used the same forms to file the costs of the outbreak at the NZa and therefore these forms give a good overview of the costs associated with an outbreak with MRSA nationwide. As far as we know this is the first study to calculate a median and range for the costs of MRSA in LTCF. Limitations are the small sample size of the on-site audit, which could have influence on the comparison with the national data. With a sample size of five in the on-site audit group, it was only possible to compare these five outbreaks with the reimbursement forms from the same outbreaks in the national data. The comparison showed that there is a trend towards a significant difference (p = 0.068). Overall, it looks like the outbreaks included in the on-site audit last longer, include more positive patients and are more expensive compared to the outbreaks included in the national data. The levels of Charlsson comorbidity index from the LTCF have not been compared. It is possible that there is a difference, however the impact on the costs of an outbreak will be small since this study looks at carriers and not at infections. There are several risk factors for the spread of microorganisms, like the use of antibiotics, but these are not included in the Charlsson comorbidity index. Furthermore, there is a potential bias regarding the on-site audit. Facilities were asked to join the audit and were not randomly selected. It is plausible that these facilities had their records in order and knowingly aimed for a higher outcome, or vice versa.

In principle, all facilities follow the national infection prevention protocols for management of MRSA in LTCF [31–33]. Most certainly, these protocols are not all implemented in the same way. One facility could, for example, have a lot more infection prevention measures in place than another one, which influences the total costs of an outbreak. Unfortunately, the outcome “death” has not been considered in the analysis, because this was not included in the reimbursement forms. Therefore, the exact number of patients who died because of an outbreak is not known. Furthermore, it is not known how facilities filled out the reimbursement forms. During the interviews for the on-site audit, some respondents informed us that they did not mention certain costs on the NZa form because it did not fit into a category. Other facilities divided these costs over other categories that were in the form. Some costs that did not fit in the reimbursement form, have been included in the on-site audit. This explains the trend towards a significant difference between the national data and the on-site audit. Because there is a financial incentive to submit a reimbursement form, we assume the dataset includes all outbreaks in the Netherlands. Therefore, we feel it is acceptable to calculate a median and range for the costs of MRSA outbreaks.
